# Life expectancy and years of life lost in HIV patients under the care of BandarAbbas Behavioral Disorders Counseling Center

**DOI:** 10.3126/nje.v7i4.20627

**Published:** 2017-12-31

**Authors:** Halimeh Yaghoobi, Hassan Ahmadinia, Ziba shabani, Reza Vazirinejad, Reza Safari, Roozbeh Shahizadeh, Fatemeh Zolfizadeh, Mohsen Rezaeian

**Affiliations:** 1 MSc Student in Epidemiology, Social Determinants in Health Promotion Research Center, Hormozgan University of Medical Sciences, BandarAbbas, Iran.; 2 PhD Student in Biostatistics, Department of Biostatistics, Hamadan University of Medical Sciences, Hamadan, Iran.; 3 Associate professor of infectious diseases, Immunology of Infectious Diseases Research Center, Rafsanjan University of Medical Sciences, Rafsanjan, Iran.; 4 Professor, PhD of Epidemiology, Social Determinants of Health Research Centre, Rafsanjan University of Medical Science, Rafsanjan, Iran.; 5 MD, Province Health Center and Research Deputy of Hormozgan University, Hormozgan University of Medical Sciences, BandarAbbas, Iran.; 6 Expert Professional Management Services for Disease Prevention. Deputy of Hormozgan University of Medical Sciences, BandarAbbas, Iran.; 7 MSc in Health Care Management, Mother and Child Welfare Research Center, Hormozgan University of Medical Sciences, BandarAbbas, Iran.; 8 Epidemiology and Biostatistics Department, Rafsanjan Medical School, Rafsanjan University of Medical Sciences, Rafsanjan, IR Iran.; 9 Occupational Environmental Research Center, Rafsanjan University of Medical Sciences, Rafsanjan, IR Iran.

**Keywords:** Life Expectancy (LE), Average Years of Life Lost (AYLL), HIV/AIDS patients, CD4 count

## Abstract

**Background:**

HIV epidemic is mostly targeted adults and has numerous negative health, social, economic, cultural and political consequences. In this study Life Expectancy (LE) and Average Years of Life Lost (AYLL) in HIV/AIDS patients are estimated.

**Materials and Methods:**

In this descriptive study all the patients at the age of 18 and more under the care of BandarAbbas Behavioral Disorders Counseling Center (BBDCC) during 2005-2015 are included. The town of BandarAbbas is center of Hormozgan Province in southern Iran. LE and AYLL have been estimated based on Life Table.

**Results:**

One hundred thirty four of the 426 eligible patients died during the study period. Compared to the general population LE for HIV/AIDS patients at age 20 is 46 years less in comparison with the general population of BandarAbbas. Moreover, a total of 8839 years of life lost during 2005-2015.

**Conclusion:**

LE in HIV/AIDS patients is less than LE among BandarAbbas general population and AYLL among them is more than general population. Most of the years of life lost are preventable if the health care system seriously will implement programs to control HIV/AIDS.

## Introduction

Life Expectancy (LE) is considered as a key measure of the illness burden which helps policymakers to allocate financial resources [1-2]. LE in a particular age is an index to calculate the average of excess years which somebody lives afterwards. The basis of computing the LE is based on the rate of specific-age mortality of the society in which the person lives [3]. Predictions of LE are considered as consequential parts of the public policy which are based on continuity of programs like social security and the health insurance [4].

Nowadays, the HIV (Human Immunodeficiency Virus) and AIDS (Acquired Immune Deficiency Syndrome) epidemics are considered as the most serious threats for the public health [5]. Therefore, some studies have shown the indirect relationship between the prevalence of HIV and LE among the population [6].

The major immunity cells which damage gradually by HIV are a group of white blood cells, namely CD4 [7]. Measuring the CD4 cells count in the blood is an important index regarding the progress of illness and death in HIV-stricken people. As a result, using the prophylaxis of opportunist viruses and supervising the therapy are considered as the essential tools to assess the qualification of individuals to begin the treatment [8-9]. 

Results of randomized trials have indicated that treatment of the disease improves the chance of survival in its advanced stage i.e. CD4count <200 [10]. According to the last report of WHO (World Health Organization), 35 million people suffer from HIV worldwide with an upward trend [11-12]. Countries in the South of the African dessert have the serious HIV prevalence and nearly 70 percent of HIV-stricken individuals of the world live there [12]. 

Presently, there is not an effective treatment for the AIDS [13, 5]. However, decreased complications and mortality due to HIV and the long-term survival of patients from the beginning of the combined treatment, ART (Anti-Retroviral Therapy), is obvious [13-18]. 

Providing Strong and advanced estimates of expected mortality among patients, as well as the continuity of anti-HIV drugs and medicinal strategies are important for the disease control. Such estimates will help the policymakers and health care planners to monitor the treatment efficiency among the population [19]. 

Quantitative measures of the improvement based on two indices i.e. LE and Average Years of Life Lost (AYLL) after diagnosis using reliable surveillance data have been calculated for other diseases but infrequently estimated for HIV/AIDA. The AYLL is estimated the average time that an individual would be expected to lives and does not die prematurely [1]. Since AIDS is a prevalent disease among youth, so its effect on the potential years of life lost is most apparent [20].

Studies show that the demolitions of LE achievements in many countries are considered as major social influences of AIDS [21]. A cohort study in England has reported that at the beginning of the treatment, the expected age of death among 35 years male patients with CD4 count less than 200, between 200 and 349 and more than 350 was estimated 71, 78 and 77 years, respectively, while LE among men in the general population of England was 78 years. After 5 years of treatment period, the expected age of death among 35 years male patients shifted to a range between 54 to 80 years [22].

Another study accomplished in the U.S reported that, LE among healthy 33 years individuals with behavioral attributes similar to HIV positive patients was anticipated 34.58 years; again the number of years lost life of the HIV-stricken individuals was estimated 11.92 years. Moreover, 2.6 and 0.7 years of lost life are due to delay in beginning and unreasonable discontinuation of antiretroviral therapy, respectively [23].

There is only one similar population-based retrospective cohort study in Iran which was accomplished on HIV-stricken patients in Isfahan Consultation Center of Behavioral disease which reports that, LE of 20 years patients was 36 years less than the general population of Isfahan. IV Drug addicts had the minimum life expectancy among other ways of infectious transmission and LE of patients with CD4 count<200 than other CD4 count rates was minimum. AYLL at the age 64 among groups of ways of infectious transmission by sexual contact and injection addiction were 39 and 39.5 years, respectively [24]. 

Given that there is not any study to estimate LE and AYLL among HIV positive patient in Hormozgan Province of Iran; as a result, the chief aim of the current study is to estimate LE and AYLL among HIV positive patient in BandarAbbas i.e. the center of Hormozgan Province of Iran. It is worth emphasizing that the number of HIV/AIDS positive cases in Hormozgan province has been increased from 523 individuals in 2004 to 1361 cases in 2015. Furthermore, the prevalence of the diseases in Hormozgan province compared to the whole country has been doubled. Since BandarAbbas has the highest number of immigrants and also has the largest behavioral disorders counseling center within the province we limit our research to this city. 

## Methodology

### Study design and the participants:

**Figure 1:**
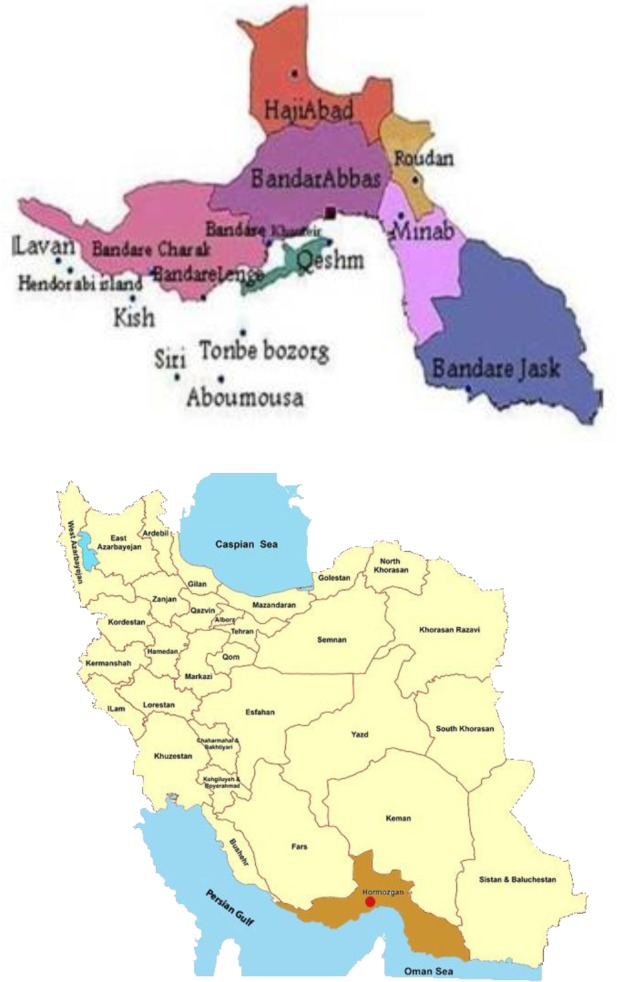
Location of BandarAbbas in the map of Iran

The current study is a descriptive one. The population under study was the HIV patients and the general population of BandarAbbas. According to the census of population in 2011 this town has 588288 people that consists of 51% male and 49% female [25]. Figure 1 shows position of BandarAbbas in Iran's geographical map.

### Sample size calculation:

Data collection was conducted by census method so that from HIV-positive patients who were under the care of BandarAbbas Behavioral Disorders Counseling Center (BBDCC), the patients who were eligible for inclusion were selected.

### Data collection:

Information gathering tool was a checklist that set up by the research group. The necessary information was extracted from patient's care file and recorded in checklist. The checklist includes demographic characteristics (age, gender, marital status, education level, occupation, time of diagnosis, the time of first visit to the Center, in case of death: time and cause of death), the basic background information (risk factors, addiction and prison history) and clinical care information (disease stage, CD4 count and beginning of antiretroviral therapy).

### Inclusion Criteria:

The criteria for inclusion into study was persons of 18 years old and above infected with HIV, which has been tested by two positive tests of Eliza and then one positive test of Western blot [26], being under care of BBDCC during period of 11 years (from 2005 to 2015).

### Exclusion criteria:

The only criterion for exclusion of the study was patients under 18 years of age.

### Outcome Variable:

Calculating LE: to calculate LE the Abridged Life Table was used [27]. Composing the life table for a community is possible with awareness of population size and the number of deaths in each age or age group during a period of time [1]. With composing the life table for the population of HIV/AIDS patients and the general population city of BandarAbbas in each separate year [28], LE of patients was compared with the general population.

Calculating AYLL: To calculate AYLL must first the years of life lost (YLL) obtain. The method of YLL calculation is as follows:

YLL = (LE for beginning of the birth - LE for each age group) × number of deaths in each age group

Then the amount of AYLL is achieved by dividing the sum of YLL (SYLL) on total deaths in that population (AYLL = SYLL ÷ Sdi) [1].

The software Life Table Designing in Excel version 2007 [29, 30] was used to calculate LE and AYLL.

Furthermore, to compare LE and AYLL in each subgroup and according to the nature of the variables paired and repeated measurement tests were used.

### Explanatory variable:

The variables studied in patients were divided into 8 category that include: 1. age group: four groups of 18-34, 35-44, 45-54, +55 years, 2. gender: two groups of male, female, 3. Education level: two groups of illiterate/elementary/guidance, high school/university, 4. marital status: three groups of married, single, divorced, 5. employment status: two groups of employed, unemployed, 6. risk factors: six groups of taking injectable materials, unsafe sex relationship with a non-homosexual, sex relationship with a homosexual, born from a suffering mother, occupational exposure, wife of a person who has one of the risk factors, 7. CD4 cell count (cells/mm three groups of under 200, 200-349, 500 and higher, 8. Beginning of antiretroviral therapy: two groups of yes and no.

### Ethical committee approval:

The Research Ethical Committee of Rafsanjan University of Medical Sciences approved the study with a certificate of approval code IR.RUMS.REC.1395.61. Due to the confidentiality of the information recorded in patient files the checklists were completed by staff of the Behavioral Disorders Counseling Center and instead of patient's name, an identification code was written.

### Data management and statistical Analysis:

The analyses were carried out applying the SPSS statistical software version 20. In all analyses P value equal or less than 0.05 is considered as significant.

## Results

In the current study, the set of collected data include the information of 431 patients with HIV/AIDS of whom 5 people (1.16%) were eliminated from the collected data due to being under 18 years. Thus, the data analysis of 426 patients (98.8%) was carried out. During the study period 134 death events (31.4%) were recorded. Demographic information of the patients is represented in Table 1.

Based on the estimation the average weight of LE in general population from 2005 to 2015, LE at birth for the general population of BandarAbbas was 69 years, and for the population of 20 years old was 50.8 years, and for the population of 35 years old was 36.8 years. However, in patient population, the LE was reported 4.8 years and 3.1 years among patients aged 20 and 35 years, respectively. Therefore, patients’ LE with 20 years old and with 35 years old was estimated to be 46, and 34 years less than the general population, respectively.

Table 2 represents LE and AYLL in HIV/AIDS 35 year old patients based on the study variables and comparing them with the general population.

Further analyses have shown that LE among the female patients is significantly higher than the male patients (P=0.011). Moreover, AYLL among the female patients is significantly less than male patients (P=0.011). LE among the patients with CD4 count<200 was less than other counterpart subgroups; but, according to the test results, the difference was not statistically significant (P=0.116). Additionally, AYLL among the patients with CD4 count<200 was higher than other counterpart subgroups; but the difference was not statistically significant (P=0.116).

LE among subgroup of the patients with sexual contact is lowet than other counterpart subgroups; but, according to the test results, the difference was not statistically significant (P=0.253). In addition, AYLL among the patients who were both injecting drug user and having sexual contact was less than other counterpart subgroups; but, the difference was not statistically significant (P=0.265). LE in the subgroup of married patients was higher than other strata of the marital status variable. However, the difference was not statistically significant (P=0.233). In addition, AYLL among the subgroup of divorced patients was higher than the subgroups of single and married patients; but, the difference was not statistically significant (P=0.233). LE among the subgroup of patients with high school/academic education is higher than the subgroup of illiterate/elementary/guidance patients; but, the difference was not statistically significant (P=0.139). Furthermore, AYLL among the subgroup of patients with high school/academic education was less than the subgroup of illiterate/elementary/guidance patients; but, the difference was not statistically significant (P=0.139). LE among the subgroup of employed patients is higher than the subgroup of unemployed patients; but, the difference was not statistically significant (P=0.244). Besides, AYLL in the subgroup of employed patients was less than the subgroup of unemployed patients; but, the difference was not statistically significant (P=0.240).

**Diagram 1:**
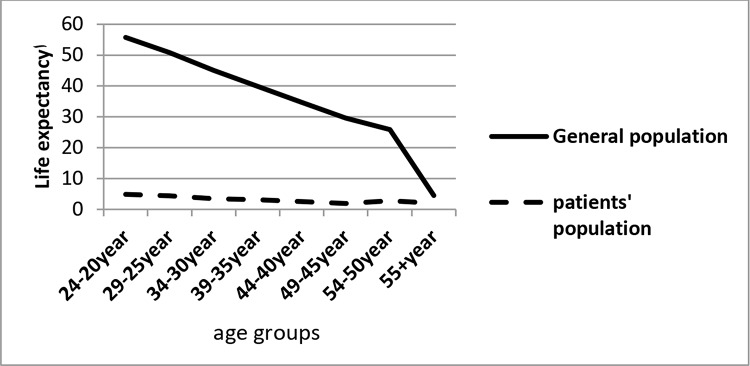
LE of HIV/AIDS patients and general population according to different age groups, BandarAbbas, Iran, 2005-2015

**Diagram 2:**
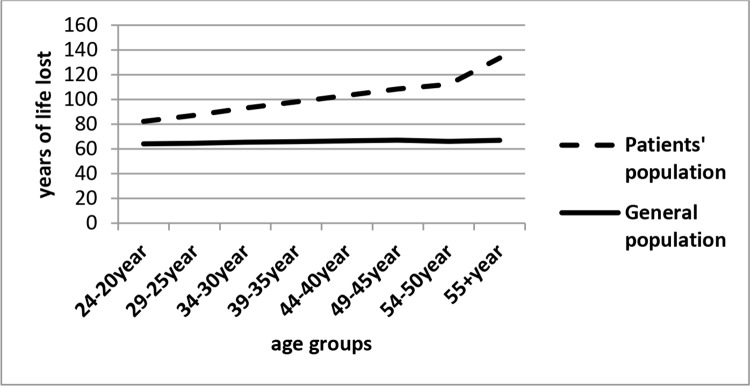
AYLL in HIV/AIDS patients and general population according to different age groups, BandarAbbas, Iran, 2005-2015

Finally, diagram 1 and 2 demonstrate the trends of LE and AYLL in patients and general population based on different age groups, respectively.

**Table 1:** HIV/AIDS Patients’ demographic characteristics, BandarAbbas, Iran, 2005-2015

Variable		N(%)
Age (years) by the time of diagnosis	Median (IQR)*	36 (10)
	18-34 years old	182 (42.7)
	35-44 years old	183 (43)
	45-54 years old	51(12)
	Above 55 years old	10 (2.3)
Gender	Male	311 (73)
	Female	115 (27)
Marital status	Married	210 (49.3)
	Single	161 (37.8)
	Divorced	55 (12.9)
Educations	Illiterate/Elementary/Guidance	342 (80.3)
	High school	79 (18.5)
	Advanced	5 (1.2)
Occupation	Employed	153 (35.9)
	Unemployed	273 (64.1)
Transmission of the disease	Sexual contact	63 (14.8)
	Injecting drug user	135 (31.7)
	Sexual contact and Injecting drug user	138 (32.4)
	Other	90 (21.1)
Stage of the disease**	Stage I	225 (56.8)
	Stage II	24 (6.1)
	Stage III	98 (24.7)
	Stage IV	49 (12.4)
CD4 cell count (cells/mm)**	Median (IQR)*	462(565.3)
	<200	92 (23.2)
	200-499	119 (30.1)
	>=500	185 (46.7)

* Inter quartile range **CD4 data was not available to 30 patients

**Table 2:** LE and AYLL in HIV/AIDS 35 year old patients based on the study variables, and making a comparison with the general population, BandarAbbas, Iran, 2005-2015

	Variable	No. of patients	No. of dead patients	AYLL in the person with 35 years old	LE in the person with 35 years old
Gender	Male	311	113	66.22	2.77
	Female	115	21	63.56	5.43
Education	Illiterate/elementary/guidance	342	115	65.92	3.03
	High school/Academic	84	19	65.45	3.54
Marital status	Married	210	65	65.53	3.46
	Single	161	50	65.9	3.09
	Divorced	55	19	66.55	2.44
Occupation	Employed	153	42	65.01	3.98
	Unemployed	273	92	66.26	2.73
Transmission	Sexual contact	63	15	67.03	1.96
of disease
	Injecting drug user	135	58	66.19	2.8
	Sexual contact and Injecting drug user	138	39	65.72	3.27
	Other	90	22	65.32	3.67
CD4 cell	<200	92	33	66.33	2.66
count (cells/mm)*
	200-499	119	29	65.43	3.56
	>=500	185	53	65.22	3.77
Total	Population of patients with HIV/AIDS	426	134	65.89	3.1
	General population of BandarAbbas	-	-	32.2	36.79

* CD4 data was not available to 30 patients

## Discussion

### Life expectancy and Average years of life lost

The main finding of this study was that people with HIV/AIDS compared to general population have substantially less LE. LE of these patients at age 20 is 4.8 years and AYLL in them is 66 years. This indicates that LE in patients with age 20 years was 46 years less than the general population and AYLL in patients was 14 years longer than the general population. These findings clearly reflect the adverse impact of HIV/AIDS on human lifetime.

The main finding of this study was that people with HIV/AIDS compared to general population have substantially less LE. LE of these patients at age 20 is 4.8 years and AYLL in them is 66 years. This indicates that LE in patients with age 20 years was 46 years less than the general population and AYLL in patients was 14 years longer than the general population. These findings clearly reflect the adverse impact of HIV/AIDS on human lifetime.

One of the factors that increased mortality amongst patients with HIV/AIDS is delayed diagnosis of the disease. At the same time, yet there are still lots of patients who take care at an advanced stage of disease. People who are diagnosed in advanced stages of disease or rejected the treatment until CD4 count of them reached below 200, they have less LE [33]. 

According to estimate conducted in our study, patients who have been diagnosed late or have been late in starting treatment so that CD4 count of them reached below 200, they have a LE at the age 20 that is at least 4 years less than patients who have CD4 count between 200 to 499. Accordingly, obvious effect of CD4 count on LE shows that the timely recognition of HIV in the early stages of the disease is important. Both patients and the health care system benefit from timely diagnosis of the disease, because patients will experience longer life and health care systems will have reduction in cost of hospital treatment [1].

### Gender

Another result of this study is that each of the indicators of LE and AYLL in male and female patients is different, so that women have more LE and less AYLL compared with men. In a study conducted in America, it was reported that AYLL in female patients is more than men [1]. In a study conducted in California, it was announced that although the patients’ LE in women is more than men, this difference was not statistically significant [32]. In a study conducted in North America and Europe, the mortality in women patients after treatment was estimated to be less than men [34]. In a study conducted on data collected from 20 cohorts of HIV-positive patients under treatment across Europe, it has been reported that the risk of death in female patients is less than male patients [18]. According to studies conducted in England, LE in female patients has been more than male patients [2].

A study conducted in America has announced AYLL at ages 20, 40, 60 and 80 years in HIV-positive women is more than men. The survey results also showed that LE of young women (under 40 years at HIV diagnosis) was less than that of males that were the same age at HIV diagnosis, but this pattern reversed in older age groups, where females who were 40 years or older at HIV diagnosis had slightly better LE than men who were diagnosed at same age. Men with infection related to male-to-male sexual contact (MSM) had longer LE than men with infection attributed to other causes, on the other hand, because a large population of HIV-infected MSM were diagnosed at a young age, so better LE among males in the younger age groups has been observed in this study. This explanation is further supported by the fact that when comparison across sex are made within the transmission methods common to both sexes, such as injecting drug use and heterosexual contact, women have longer life expectancy than men [31].

In general, higher LE in female patients than men may be due to the fact that early detection of disease is done through screening during pregnancy in care centers before mother reaches to low CD4 count. Late presentation in men to medical centers, large gender differences in lifestyle factors in patients e.g., alcohol and drug abuse smoking, is more frequent in men. On the other hand, bias caused by the lack of follow-up and result of proving death in women can exist. To further understand the differences, it is important we formulate policy to ensure equal opportunities for men and women to achieve the same results [2]. Variation in the level of education, level of income, social stigma, access to health care systems and isolation of patients are other features that may influence the gender differences observed in the HIV/AIDS patients [35].

### Injecting drug use

In our study, injecting drug users than other disease transmission methods had the worst LE and AYLL, but these differences were not statistically significant. A Canadian study reported that people with a record of injecting drug use have a higher mortality rate and low LE [11]. The results of a cohort study conducted in North America reported that there is a significant difference in LE of HIV transmission method group, so that the low LE in all periods for people with a record of injecting drug use has been observed [35]. In a study conducted in Isfahan, similar results with our findings have been reported, despite the fact that LE in patients with a record of drug abuse was lower, but there was no significant difference between this and other disease transmission methods [24]. In a study conducted in America during the 10 year period, injecting drug users in men and women with HIV have worst LE among all classes of disease transmission method [1].

The high mortality in people with HIV/AIDS injecting drug users observed in the literature was continually reported. Poor socio-economic status, reduced access to health care services, co-infection with hepatitis C infection and tuberculosis and housing instability are the causes of reducing LE in this population [35,18,11]. Probably the low sample size is why this finding was not significant in our study.

### Job, Education and Marital status

The difference between LE and AYLL in “employed and unemployed” patients, “illiterate/elementary/guidance and high school/university” patients and “married, single or divorced” patients was not statistically significant in this study. In a study conducted in Isfahan, the difference for the education level of the patients was not statistically significant, but this difference was significant about job and marital status [24]. A study conducted in America has announced an increase in mortality for diseases in singles and divorcees than the married people [36].

Risk behaviors in people with low education, low socioeconomic status as well as in unemployed and needy people who financially have delinquent behavior are more observed [21]. People with more sexual partners may be at higher risk of contracting sexually transmitted diseases, including AIDS than those who have reliable sexual partners. It is possible that single people and divorced individuals have a wider sexual network which raises their risk of obtaining HIV/AIDS. It is also likely that there would be a weak social solidarity among single and divorced people than the married people that leads to higher death following the disease in these groups [36].

### CD4

Another finding of this study was that although the worst LE and AYLL in patients with CD4 count <200 has been observed relative to the other subgroups counterpart this difference was significant from the statistical point of view. In the survey conducted in Isfahan and Colombia, similar result with our study finding has been reported [24, 37]. Results announced by a study conducted in the United States are so that those who have started the treatment with CD4 count above 500 had a LE 9 years more compared to those treated in the CD4 count below 350 [10].

One of the most important factors in reducing LE in people with HIV in comparison with the general population is that patients have begun the treatment in low CD4 count position relative to the recommend instructions. At the beginning of treatment, mortality for those who have the lowest CD4 count is higher and even CD4 count at the start of antiretroviral treatment remains as prognosis of death in those who have survived at first three years of treatment. Patients, especially those who have no symptoms, may be delays in starting treatment, because they are reluctant to invest in a lifetime dedication to antiretroviral drug consumption [2].

### Age group

In our study, it was found that the average index in different age groups between patients and the general population is significantly different on each of the indicators of LE and AYLL. In the survey conducted in London, it was reported that there is a strong evidence of the impact of age on HIV/AIDS disease due to change of antibodies, so that there is a clear gradient of increased mortality risk with increasing age (especially age 45 and older) [18]. A number of other studies that have compared deaths in people with HIV and healthy subjects have also found that there is a significant death rate with increasing patient age compared to non-infected population [38, 39].

## Conclusion

According to BBDCC data; during 2005-2015, LE in HIV patients is less than LE among BandarAbbas general population, so that at the age of 20, the patients’ LE is 46 years less. AYLL in HIV/AIDS patients is also 14 years more than general population. Most of the years of life lost are preventable if the health care system will seriously implement programs to control HIV/AIDS.

### Limitation of the study:

The limitation of our study was the incompleteness of demographic and laboratory information registered in care forms. For solving this problem, the incomplete information was filled through making phone contact with the patient by counseling center’s personnel, otherwise the patient’s record was deleted from the study; 8 files containing incomplete information were deleted from the study. 

### Future scope of the study:

Further longitudinal research is needed to determine what factors will have more impact on LE of patients with HIV/AIDS.

### What is already known on this topic?

Life span assessment of patients with HIV/AIDS is already carried out in some parts of the world.

### What this study adds:

According to BBDCC data; during 2005-2015, LE in HIV patients is less than LE among BandarAbbas general population, so that at the age of 20, the patients’ LE is 46 years less. AYLL in HIV/AIDS patients is also 14 years more than general population. 

## Acknowledgements: 

We are thankful to Health Center, Registration Office and Information Program and Budget Office of Hormozgan province for their support. We also appreciate colleagues of BBDCC for their support. It is noteworthy that this article is the outcome of Ms. Halimeh Yaghoobi’s thesis for receiving the master degree in epidemiology from Rafsanjan Medical School.

## References

[ref1] Harrison D, Song  R, Zhang  X (2010). Life Expectancy After HIV Diagnosis Based on National HIV Surveillance Data From 25 States, United States. J Acquir Immune Defic Syndr.

[ref2] May M, Gompels M, Delpech V, Porter KH, Post F, Johnson M (2011). Impact of late diagnosis and treatment on life expectancy in people with HIV-1: UK Collaborative HIV Cohort (UK CHIC) Study. BMJ.

[ref3] Nsanzimana S, Remera E, Kanters S, Chan K, Forrest J, Ford N (2015). Life expectancy among HIV-positive patients in Rwanda: a retrospective observational cohort study. Lancet Glob Health.

[ref4] Sabin C (2013). Do people with HIV infection have a normal life expectancy in the era of combination antiretroviral therapy?. BMC Medicine.

[ref5] Olshansky J, Passaro D, Hershow R, Layden J, Carnes B, Carnes J (2005). A Potential Decline in Life Expectancy in the United States in the 21st Century. N Engl J Med.

[ref6] Mirzaei M, Poorolajal J, Khazaei S, Saatchi M (2013). Survival rate of AIDS disease and mortality in HIV-infected patients in Hamadan, Iran: a registry-based retrospective cohort study (1997–2011). Int J STD AIDS.

[ref7] Hogg R (2008). Life expectancy of individuals on combination antiretroviral therapy in high-income countries: a collaborative analysis of 14 cohort studies. Lancet.

[ref8] Yavari P. Epidemiology Textbook of Prevalent diseases in Iran. 1th ed. Tehran: GAP, 2013, pp 29-44. (Book in Persian)

[ref9] Ford M, Meintjes G, Pozniak A, Bygrave H, Hill A, Peter T, et al. The future role of CD4 cell count for monitoring antiretroviral therapy. Lancet Infect Dis. 2014; Published Online November 19, 2014 
https://doi.org/10.1016/S1473-3099(14)70896-5

[ref10] Moore R, Chaisson R (1999). Natural history of HIV infection in the era of combination antiretroviral therapy. AIDS.

[ref11] Romley J, Juday T, Solomon M, Seekins D, Brookmeyer R, Goldman D (2014). Early HIV Treatment Led To Life Expectancy Gains Valued At $80 Billion For People Infected In 1996–2009. Health Affairs.

[ref12] Patterson S, Cescon A, Samji H, Chan K, Zhang W, Raboud J (2015). Life expectancy of HIV-positive individuals on combination antiretroviral therapy in Canada. BMC Infect Dis.

[ref13] World Health Organization. Global update on HIV treatment 2013: results, impact and opportunities. [online] 2013. [cited 2017 Apr 28]. Available from: http://www.who.int/hiv/pub/progressreports/update2013/en/.

[ref14] Torkashvand F, Asadpor M, Sheikh Fathollahi  M, Sheikhi E, Salehi Shahrbabaki  M.H, Hoseini OR (2015). Frequency of High Risk Behaviour in HIV Positive Patients Referred to Centers for Behavioural Disorders of Rafsanjan and Kerman in 2012. J Rafsanjan Univ Med Sci.

[ref15] Wong KH, Chan KC, Lee SS (2004). Delayed progression to death and to AIDS in a Hong Kong cohort of patients with advanced HIV type 1 disease during the era of highly active antiretroviral therapy. CID.

[ref16] Hogg RS, Heath KV, Yip B, Craib KJ, O'shaughnessy MV, Schechter MT (1998). Improved survival among HIV-infected individuals following initiation of antiretroviral therapy. JAMA.

[ref17] Jaggy C, Overbeck J, Ledergerber B, Schwarz C, Egger M, Rickenbach M (2003). Mortality in the Swiss HIV Cohort Study (SHCS) and the Swiss general population. THE LANCET.

[ref18] Detels R, Munoz A, Farlane G, Kingsley L, Margolick J, Giorgi J (1998). Effectiveness of Potent Antiretroviral Therapy on Time to AIDS and Death in Men With Known HIV Infection Duratin. JAMA.

[ref19] Bhaskaran K, Hamouda O, Sannes M, Boufassa F, Johnson A, Lambert P (2008). Changes in the Risk of Death After HIV Seroconversion Compared With Mortality in the General Population. JAMA.

[ref20] Sharifi Renani  H, Akhoondi N, Honarvar N, Mohammadi  M (2014). Effects of the HIV/AIDS epidemic on economic Growth in Iran. J Research Health.

[ref21] Johansen J, Smith E, Juel K, Rosdahl N (2005). The AIDS epidemic in the city of Copenhagen, Denmark: Potential years of life lost and impact on life expectancy. Scand J Public Health.

[ref22] Daryazadeh  S, Maryami  F (2013). Epidemiological Investigation of HIV-Positive Patients in Isfahan Behavioral Consultation Center, Iran. J Isfahan Med Sch.

[ref23] Maya  M, Gompelsb M, Delpechc V, Delpechc KH, Orkine CH, Kegg S (2014). Impact on life expectancy of HIV-1 positive individuals of CD4R cell count and viral load response to antiretroviral therapy. AIDS.

[ref24] Losina E, Schackman B, Sadownik S, Gebo K, Walensky R, Chiosi J (2009). Racial and Gender Disparities in Life Expectancy Losses Among HIV-infected Persons in the United States: Impact of Risk Behavior, Late Initiation and Early Discontinuation of Antiretroviral Therapy. Clin Res Infect Dis.

[ref25] Mohammadi-Moein HR, Maracy MR, Tayeri K (2013). Life expectancy after HIV diagnosis based on data from the counseling center for behavioral diseases. J Res Med Sci.

[ref26] Tabarsi P, Mirsaeidi SM, Amiri M, Mansouri SD, Masjedi MR, Velayati AA (2008). Clinical and laboratory profile of patients with tuberculosis/HIV coinfection at a national referral centre: a case series. East Mediterr Health J.

[ref27] Wunsch G, Mouchart M, Duchêne J, eds. The life table: modelling survival and death. 1th ed. Netherlands, Springer Science & Business Media: 2013 Mar 9, PP 11-25.

[ref28] Gardner JW, Sanborn JS. Years of potential life lost (YPLL)—what does it measure? Epidemiology. 1990; 1:322–9. https://doi.org/10.1097/00001648-199007000-00012

[ref29] Fereshtehnejad M, Asadi-Lari  M, Moradi Lakeh  M, Vaez mahdavi, Motvaliyan A, Afkari M (2010). Estimation of life expectancy and its association with social determinants of health in Urban population of different districts of Tehran in 2008 (Urban HEART Study). Teb & Tazkiyeh.

[ref30] Chiang CL (1972). On constructing current life tables. Am Stat Assoc.

[ref31] Siddiqi A.E, Hall HI, Hu X, Song R (2016). Population-Based Estimates of Life Expectancy After HIV Diagnosis: United States 2008–2011. J Acquir Immune Defic Syndr.

[ref32] Marcus JL, Chao CR, Leyden WA, Xu L, Quesenberry CP, Klein DB (2016). Narrowing the gap in life expectancy between HIV-infected and HIV-uninfected individuals with access to care. JAIDS.

[ref33] Nakagawaa F, Lodwicka R, Smitha C, Smithb R, Cambianoa V, Lundgrenc  J (2012). Projected life expectancy of people with HIV according to timing of diagnosis. AIDS.

[ref34] Antiretroviral Therapy Cohort Collaboration. Mortality of HIV-infected patients starting potent antiretroviral therapy: comparison with the general population in nine industrialized countries. Inte J Epidemiol. 2009; 38:1624-33 https://doi.org/10.1093/ije/dyp306

[ref35] Samji H, Cescon A, Hogg R, Modur SH, Althoff K, Buchacz K (2013). Closing the Gap: Increases in Life Expectancy among Treated HIV-Positive Individuals in the United States and Canada. PLOS One.

[ref36] Kposowa AJ (2013). Marital status and HIV/AIDS mortality: evidence from the US National Longitudinal Mortality Study. Int J Infect Dis.

[ref37] Lima VD, Hogg RS, Harrigan PR, Moore D, Yip B, Wood E (2007). Continued improvement in survival among HIV-infected individuals with newer forms of highly active antiretroviral therapy. Aids.

[ref38] Lohse N, Hansen AB, Pedersen G, Kronborg G, Gerstoft J, Sørensen  HT (2007). Survival of persons with and without HIV infection in Denmark, 1995–2005. Ann Intern Med.

[ref39] van Sighem  A, Danner S, Ghani AC, Gras L (2005). Anderson RM. de Wolf F. Mortality in patients with successful initial response to highly active antiretroviral therapy is still higher than in non-HIV-infected individuals. J Acquir Immune Defic Syndr.

